# Medical Center Farmers Markets: A Strategic Partner in the Patient-Centered Medical Home

**DOI:** 10.5888/pcd10.130105

**Published:** 2013-08-01

**Authors:** Daniel R. George, Liza S. Rovniak, Jennifer L. Kraschnewski, Kathy J. Morrison, Judith F. Dillon, Beth Y. Bates

**Affiliations:** Author Affiliations: Liza S. Rovniak, Jennifer L. Kraschnewski, Kathy J. Morrison, Judith F. Dillon, Penn State Hershey College of Medicine, Hershey, Pennsylvania; Beth Y. Bates, Penn State Hershey College of Nursing, Hershey, Pennsylvania.

## Abstract

**Background:**

The number of medical center–based farmers markets has increased in the past decade, but little is known about how such organizations contribute to the preventive health goals of the patient-centered medical home.

**Community Context:**

In 2010, we started a seasonal farmers market at Penn State Hershey Medical Center to help support the institution’s commitment to the medical home.

**Methods:**

We obtained descriptive data on the farmers market from hospital and market records and tracking information on the market’s Facebook and Twitter sites. We computed summary measures to characterize how the market has begun to meet the 6 standards of the 2011 National Committee for Quality Assurance’s report on the medical home.

**Outcome:**

During the 2010 and 2011 seasons, 146 medical center volunteers from 40 departments formed 23 interprofessional teams that spent an average of 551 volunteer hours per season at the market, providing health screenings (n = 695) and speaking to customers (n = 636) about preventive health. Fifty-five nonmedical community health partners provided 208 hours of service at the market alongside medical center staff. Market programming contributed to 5 regional preventive health partnerships and created opportunities for interprofessional mentoring, student leadership, data management, development of social media skills, and grant-writing experience. The market contributed to all 6 medical home standards outlined by the National Committee for Quality Assurance.

**Interpretation:**

Medical center markets can support medical home standards. With systematic tracking of the health effects and integration with electronic medical health records, markets hold potential to contribute to comprehensive patient-centered care.

## Background

Patient-centered medical homes (PCMHs) are increasingly prevalent in the United States; more than 7,600 clinicians and 1,500 practices have earned PCMH recognition as of 2011 (1). As adoption of the PCMH has spread, the US Department of Health and Human Services has developed a standard definition of a medical home. This definition encompasses 5 attributes — patient-centeredness, comprehensive care, coordinated care, access to care, and a systems-based approach to quality and safety (2). The National Committee for Quality Assurance (NCQA) has sought to establish 6 national standards and performance measures: 1) enhance access and continuity; 2) identify and manage patient populations; 3) plan and manage care; 4) provide self-care support and community resources; 5) track and coordinate care; 6) measure and improve performance (3). The ultimate goal of the PCMH model is to achieve whole-person care, leading to more effective and efficient outcomes and improving patient satisfaction while lowering long-term costs (4–9). Because such comprehensive care often cannot occur in a single primary care visit, the PCMH model encourages strategic partnerships to strengthen the abilities of medical centers and clinics to provide integrated care (10,11).

Farmers markets at medical centers represent one such unique partnership that can contribute to the preventive health goals and NCQA standards for a PCMH. Medical center–based markets have increased exponentially over the past decade (12–14). Of the 7,864 operational markets in the United States in 2012 (15), 91 markets exist exclusively on medical campuses (12). As recurrent organizations at fixed locations where vendors sell farm products and other goods, markets are attractive assets in any community, but they are especially relevant for the rising number of medical centers committed to the PCMH.

In addition to increasing access to healthful products, medical center farmers markets can contribute to the PCMH by providing services to help alter dietary and lifestyle choices, including health screenings, cooking demonstrations, physician “prescriptions” to purchase healthy produce, and information on lifestyle-change programs. Furthermore, medical center–based markets can furnish a sustainable supply of interprofessional staff to provide such services — including medical center volunteer staff, student interns, and health professionals interested in continuing education opportunities. Because such markets typically operate at least 6 months per year (and could operate year-round in milder climates and indoors), they can contribute to building long-term links with health care providers to promote comprehensive patient-centered care. Furthermore, public health services and programs provided by medical center markets can contribute to core values of the PCMH model, including sensitivity to context and cultural differences, interdisciplinary collaboration, information management, community engagement, and access to care. Forging service-learning experiences outside of the clinical setting and in community venues such as markets is crucial to educating medical personnel and trainees about local needs and systems-based factors necessary to address both acute patient care and larger public health issues (16). Although the PCMH movement has made interdisciplinary partnerships among health professionals a priority (17), to date, no study has described how health provider partnerships among the growing number of medical center markets can contribute to the PCMH model.

### Community Context

At Penn State Hershey Medical Center (PSHMC), we have used our farmers market, which has operated on campus for 2 years, to advance PCMH preventive health goals while developing continuing education opportunities for health professionals and trainees within an organization that has recently transitioned to the PCMH. In response to local needs assessments indicating high rates of obesity and sedentary lifestyles (18), market leadership has developed the market to target childhood obesity, cardiovascular disease, stroke, and women’s health. In this article, we provide a descriptive overview of how preventive health programming developed at our market has addressed the NCQA’s 6 standards (3). We also provide a logic model to guide development of similar efforts at medical centers that are transitioning to the PCMH model and have the capacity to support famers markets.

## Methods

### Descriptive overview of the market

In 2010, we launched a weekly farmers market open seasonally on Thursdays from May through October on 10,000 square feet of farmland surrounding the PSHMC campus. The vision for the market was to combine agricultural, medical, and community resources to contribute to the long-term health of the region by providing more comprehensive care and services for patients and families. The market provides 25 vendors offering locally produced fruits and vegetables (5), organic dairy products (2), free-range meats (1), whole-grain baked goods (1), and assorted specialty items (16). It is registered with the federal Supplemental Nutrition Assistance Program (SNAP) and accepts food stamps. In its 2 seasons, the market has averaged approximately 350 customers per week and 7,500 customers per season, with 68% of customers being medical center employees, 22% being community members, and 10% being patients and families. It is run by a volunteer director, a paid part-time manager, and a team of 8 volunteers from PSHMC and the community who handle market operations and logistics, scheduling, marketing and social media outreach, and preventive health programming (12).

### Initial needs assessment

Prior to the market’s opening, and in accordance with our medical center’s transition to the PCMH, the nursing community outreach team conducted a community health needs assessment of the region by reviewing the focus areas identified in *Healthy People 2010* (18) and by collaborating with nursing colleagues from the Pennsylvania Department of Health. Results indicated that local health needs were consistent with national needs as reported in *Healthy People 2010* (19). The medical center saw an opportunity to address the growing issues of cardiovascular disease, stroke, and women’s health and nutrition through the market, the customer base for which is approximately 70% women under age 40. Also, a children’s hospital on campus offered the opportunity to address childhood obesity.

### Partnership building

Working from the shared belief that a medical center’s mission is not only to treat illness reactively but also to proactively promote health for patients, employees, and the community — a concept consistent with the PCMH model implemented at PSHMC in 2008 — the nursing community outreach team worked with the market director (D.R.G.) to forge strategic partnerships between the market and health professionals in areas such as medicine, public health, and nutrition. A permanent “preventive health” booth within the market was established for addressing community health needs.

To capitalize on the expertise and knowledge within the academic medical center and to address the PCMH focus on interdisciplinary teamwork, the nursing community outreach team invited multiple Penn State health professionals and trainees to volunteer at the market. Managers of 60 departments were contacted, and managers whose resources and expertise matched the needs assessment were given priority. Invitations requested that volunteers join with nursing volunteers to provide health screenings and to develop educational topics that addressed *Healthy People 2010* priority areas (eg, heart health, child safety, women’s health). Professionals and students from more than 40 disciplinary backgrounds signed up to volunteer for initial market sessions and submitted programming ideas.

### Long-term health outreach

We aimed to combine healthy lifestyle programming with nutritious market offerings in a way that would add greater value than traditional health fairs could. Although health fairs are the most recognized form of community-based health promotion in the United States, the lack of continuity and appropriate follow-up may limit their effectiveness. We believed the recurrent presence of interprofessional teams involved in one-on-one teaching with interactive tools at a weekly market could enhance PSHMC’s mission to serve as a community venue for preventive health promotion and whole-person care (1). By operating 6 months per year, the market offers a longer period for intervention and relationship building than health fairs and is connected to a medical system operating year-round.

The nursing department began developing a plan with the market director to provide weekly preventive health-screenings (based on the concept that many people do not know their status for blood pressure, pulse, body mass index, cholesterol, blood glucose, or adherence to exercise guidelines) (20), interdisciplinary one-on-one teaching with customers, handouts with action plans for healthy improvements, and a weekly health education topic. In recognition of the importance of nutrition and behavior modification for improving health-screening numbers, the Center for Nutrition and Activity Promotion was asked to partner with nurses to conduct screenings. Nutritionists designed educational resources (eg, guidance on how to read food labels and manage weight) that complemented the screenings and provided a staff member each week to address customers’ questions on nutrition. Weekly market programming was promoted through a Facebook page, Twitter account, and e-mail newsletter.

To measure the impact of the market in meeting education and PCMH goals, we obtained descriptive data from hospital and market records and tracking information on the market’s e-mail listserv and its Facebook and Twitter sites. We computed summary measures to characterize how the market met the 6 standards outlined in the 2011 NCQA report on the PCMH (6).

## Outcome

The market contributed to addressing all 6 standards outlined in the 2011 NCQA report.

### Standard 1: Enhance access and continuity

The Facebook and Twitter networks and the e-mail listserv have amassed a combined 4,000 followers, who receive daily market updates and evidence-based preventive health information. Facebook and Twitter platforms are also used to engage customers in online conversations about healthier lifestyles and to inquire about community needs.

The farmers market has created a venue in which the medical center is annually able to reach a diverse demographic of nearly 10,000 community members with preventive health education during a 6-month market season. Medical center volunteers have directly engaged an average of 636 customers each season in conversations about topics such as stroke risk awareness, nutrition, and activity promotion. This weekly programming — available in English or Spanish — has enabled professionals from approximately 40 medical center departments to interact with the public and share expertise.

The market’s preventive health programming has also created an opportunity for these professionals to mentor medical and nursing students and model clinical skills in an informal setting. Having students and health professionals interacting as practice teams with the common goal of community education has the potential to improve interprofessional relationships and support teamwork and care coordination. Students get real-world practice with patients and hone clinical skills under the supervision of trained professionals. Because of scheduling issues and other barriers, students in teaching hospitals often have few opportunities for one-on-one mentoring and interprofessional engagement. Furthermore, the market creates opportunities for examining community needs and health literacy to guide refinement of preventive health programming.

### Standard 2: Identify and manage patient populations

During the 2010 and 2011 seasons, 146 medical center volunteers from more than 40 medical center departments (Box) spent an average of 551 volunteer hours at the market and provided 695 screenings. These screenings consisted of measures of routine preventive care, including blood pressure and heart rate checks, body mass index calculation, and assessment of skin cancer risk and osteoporosis. Discussions with market customers, including market vendors who lack formal health care, addressed knowledge of blood glucose and cholesterol levels and included guidance on preventing chronic disease. Customers were evaluated for lifestyle behaviors that can affect the risk for chronic disease, such as nutrition, physical activity, and tobacco use.

**Box. Volunteer Partners, Penn State Hershey Medical Center**

Clinical Institutes/Departments

  Blood Bank

  Bone and Joint Institute

  Breast Center

  Cancer Institute

  Cardiac Rehab/FitRx

  Center for Nutrition and Activity Promotion

  Child Life Department

  Children’s Hospital Pediatric Injury Prevention Program

  Clinical Nutrition Services

  Dermatology Department

  Diabetes Center

  Division of Pediatric Neurology

  Heart and Vascular Institute

  Inpatient Psychiatric Care

  Life Lion Emergency Services

  Neurology Department

  Nursing Department

  Express Admission Unit/Trauma

  • Float Pool Unit

  • Medical Intensive Care Unit

  • Neonatal Intensive Care Unit

  • Neurosciences Intensive Care Unit

  • Operating Room

  • Pediatric Intensive Care Unit

  • Perianesthesia Unit

  Obesity/Bariatric Program

  Pharmacy Services

  Stroke Program

  Women’s Health Program

  Academics

  Penn State College of Medicine

  Food as Medicine medical student group

  Humanities Department

  Penn State Hershey Library

  Penn State School of Nursing

  Community Partners

  Community Involvement Team

  Domestic/Sexual Violence Medical Advocacy Group

  Master Gardeners

  Penn State Messiah Nursing

In many instances, repeat customers visited the preventive health booth to monitor their vital signs, and volunteers documented that at least 36 people changed their blood pressure medications as a result of screenings. Building upon the success of the market-based screening and educational services, market leadership was invited to help markets in urban underserved areas in Harrisburg, Pennsylvania, set up health-screening programs modeled after the medical center market. These outreach services provided PSHMC medical and nursing students with interprofessional service-learning opportunities with low-income populations.

### Standard 3: Plan and manage care

Although the market is not clinically oriented, it has provided a platform for augmenting the care and education offered in primary care settings. The market has especially emphasized providing evidence-based preventive health guidelines and care management to at-risk and low-income patients and community members who often are underserved by traditional medical systems.

The following evidence-based care initiatives have targeted underserved populations:

- *Prevention produce/health care for low-income workers*: Medical and nursing student volunteers formed a group called “Food As Medicine” and each week purchased, cleaned, and bundled seasonal produce from market vendors and distributed it on Friday mornings to nearby low-income workers in need of fresh, healthful foods. Additionally, students developed bilingual messages written at the sixth-grade reading level about preventive health (eg, hand washing, sun protection) and bundled it with the produce. Funding for the 6-month program was established through a community organization with ties to the market. Through the collaboration, unsold market produce was used effectively, and students provided free labor while also taking advantage of opportunities in leadership, education, and professional development centered on local needs. Ultimately, market customers helped subsidize the project by purchasing produce at full price, which in turn enabled farmers to sell produce for the program at reduced cost. This partnership with low-income workers continued after the market season, enabling students and staff to engage an additional 142 adults and 14 children from the partner populations in immunization and dental-care programs. Many of the at-risk clients benefiting from medical outreach services had underlying respiratory disease and had never been immunized for influenza. The sites at which low-income workers were served have become partner sites for a first-year medical school course, “Social Influences in Health,” which requires students to make service-learning visits to underserved areas.
- *Partnership with local food banks*: Medical students delivered produce to local food banks and were invited to partner with the US Department of Agriculture’s National School Lunch Program (21) to provide and serve lunches for children in a neighborhood that qualified for the program based on need.
- *Charitable partnerships*: The market has offered ongoing support for charities that purchase and distribute produce to homeless shelters and halfway homes in central Pennsylvania.

Future goals include setting up mobile markets that would drive into rural and urban underserved areas, distribute unsold or leftover fresh produce from local vendors, increase levels of SNAP reimbursement, and provide health-screening services.

### Standard 4: Provide self-care support and community resources

The market has supported self-care by providing greater access to local, nutritious, organic foods, by distributing recipe cards to shoppers, and by holding free classes led by guest chefs on preparing healthy meals using ingredients from the market. Furthermore, the market has annually included 55 nonmedical community partners as vendors in the market; these vendors have provided 208 hours of wellness-promoting service alongside staff from PSHMC. They have offered free workshops on holistic health, Reiki demonstrations, yoga and tai chi workshops, acupuncture information, and aromatherapy, as well as information on local fitness centers, businesses, and environmental groups. Such programming builds on the PCMH goal of providing whole-person, prevention-oriented care that draws upon community resources.

### Standard 5: Track and coordinate care

Because the market is held weekly, it is an ideal place to educate community members about health status and provide chartable week-to-week information on vital signs and other metrics that can be documented and used to demonstrate progress derived from healthier lifestyles. Although our market currently tracks data on paper slips given to customers, a future goal is to collaborate with engineers to develop secure databases for charting customer data throughout the season. Such a database could enhance the market’s capability to promote preventive lifestyles among community members and support the PCMH model. When customers have been identified as high-risk for a particular condition, volunteers have referred customers to their physician and provided dietary advice that could be immediately acted on in the market through the purchase of nutritious foods. In the absence of a primary care provider, referrals for follow-up are made through the PSHMC Care Line. This process is consistent with the PCMH emphasis on primary and secondary prevention of chronic disease.

### Standard 6: Measure and improve performance

Market leadership has partnered with medical and nutrition students to conduct surveys and focus groups of market customers as well as employees and community members who do not use the market. These efforts contribute to more effective delivery of services each season. In the past 2 years, students have initiated grant applications for prevention-oriented outreach projects stemming from observations of community needs and collaborative relationships cultivated at the market. For instance, medical students wrote a grant proposal to participate in the “Prescription Produce” program that enables doctors to write prescriptions (redeemable for fruits and vegetables at local markets) for high-risk patients. Medical center administration has recognized these students by granting them institutional community service awards.

### Future research goals

Market leadership has identified multiple areas for refinement of its operations to better meet PCMH standards, including establishing more comprehensive computer databases and capitalizing on automated technology to track processes and outcomes related to PCMH priorities. Such priorities include the following:

- Enhanced health care access
- Interdisciplinary partnerships among hospital employees, students, communities, and regional health systems
- Increased cultural sensitivity
- Improvements in clinical skills
- Improvements in social media skills for advancing preventive health
- Increased understanding of community health needs
- Leadership and civic activism related to community health
- Reduced health care costs
- Improved quality of care, as measured by patient health outcomes, and patient/family satisfaction

Although our experiences suggest positive effects related to these PCMH goals, more inquiry will be necessary. We developed a logic model (Figure) based on the inputs and outputs that have contributed to the PCMH standards; the model provides guidance for market replication and improvement in other regions.

**Figure:** A logic model for how a farmers market can serve National Committee for Quality Assurance’s standards for the patient-centered medical home (3).

**Table:** 

Inputs →	Outputs →	Outcomes
**Standard 1: Enhance access and continuity**		
• Interprofessional health education teams with at least 1 bilingual English/Spanish staff member	• Provide bilingual health screening data or information	**Current outcomes**
		• Educated diverse market customers
	• Provide mentoring and patient interaction for medical, nursing, nutrition, and other students	
		• Approximately 2,200 Facebook members
		• Average number of people talking about Facebook page each week during market season: approximately 125^a ^
• Facebook page		
	• Weekly Facebook updates about health-screening services, chef demonstrations, recipe cards, market products, health services, music programming	
• Twitter profile		• Average total weekly reach of audience: approximately 5,000
• E-mail newsletter		• Approximately 150 followers
		• More than 80 “retweets” of information per season
	• Share links to nutrition and health websites	• Approximately 1,500 recipients each week during market season
		**Future goals**
	• Respond to questions and comments of customers	• Train health professionals in health promotion via social media and improve communication skills
	• Repost Facebook content	• Measure number of newsletters opened
	• Ask followers to repost (“retweet”) information	• Measure health literacy of market customers
		• Track number of people using bilingual health-screening services
	• E-mail version of weekly Facebook information	
		• Develop program to follow up with customers using telephone and electronic communication
		• Explore year-round market format
**Standard 2: Identify and manage patient population**		
• 10,000 square feet of level space for market operations	• Venue for weekly market and preventive health programming for community members, employees, and patients	**Current outcomes **
		• Provided 695 health screenings annually
		• Increased customer and vendor awareness of effect of healthy lifestyles on chronic disease prevention
• 300 square feet of devoted space within the market, including 3 tents and 4 tables		
	• Provide 551 volunteer hours annually	
		• Increased student knowledge about data management and interprofessional collaboration
	• 23 interprofessional teams interact with customers and vendors	
• 146 medical center staff volunteers from 40 departments		
	• Collect data on customer demographics and health status	• Provided opportunity for student service-learning experience through screening program in high-need area
• Screening equipment (eg, blood pressure, bone density, skin cancer)		
		**Future goals **
		• Continue expanding health-screening programming to other vulnerable regional populations
		• Track actions taken by customers after health screening
		• Track whether health-screening information was acted on by physicians
		• Track change in student clinical skills at market
**Standard 3: Plan and manage care**		
• Student volunteer “Food as Medicine” group	• Partnerships between medical center and 2 local food banks	**Current outcomes**
		• Contributed to medical center’s community service mission
• Community/grant funding	• Partnerships between medical center and 3 local charities	
		• Delivered produce and preventive health messages to dozens of low-income community members
• Immunization and dental care outreach services		
	• Students provided approximately 100 volunteer hours as part of medical student–led community service projects	
		• Delivered free influenza immunizations and dental services to 156 community members
	• Immunization and dental care staff provided approximately 10 volunteer hours	• Delivered approximately 20 healthy lunches to low-income youth
	• Provided evidence-based preventive health guidelines and care management to at-risk and low-income patients and community members who often are underserved by traditional medical systems	• Delivered approximately $1,200 of market produce to local charities annually
		**Future goals **
		• Set up mobile farmers market to deliver produce to food deserts
		• Set up database to track number of low-income people served by market and change in health status over extended period
		• Integrate prevention produce into school healthy lunch program
		• Track data on sale of produce vis-à-vis other products
		• Increase reimbursement of SNAP (Supplemental Nutrition Assistance Program) benefits at market
**Standard 4: Provide self-care support and community resources**		
• Market director, manager, volunteers	• 25 vendors offered local and organic food/health products	**Current outcomes **
		• Generated approximately $140,000 in sales of food or health products per season for local vendors
• Market vendors	• 208 hours of service by health services groups (eg, free yoga and tai chi demonstrations)	
		• Increased awareness of 55 integrative-medicine options in region
• Community health services groups		
		• Contributed to whole-person, prevention-oriented care
	• Approximately 1,000 free healthful-recipe cards distributed per season	
		**Future goals **
• Walking paths to market	• Approximately 5 chef demonstrations conducted for healthy meal preparation per season	• Provide booths and sign-up sheets for social events related to healthy living (eg, local walking/hiking/sports groups, healthy restaurant visits)
• Bicycle racks		
• Approximately 300 parking spaces		
		• Provide booth to enable preventive health researchers to test health-related intervention ideas and recruit participants for preventive health research
		• Provide booth to help researchers recruit participants for health-related studies
		• Track change in customer use of self-care resources
**Standard 5: Track and coordinate care**		
• Paper slips for test tracking and follow-up	• Make referrals for patients	**Current outcomes **
	• Follow up with referred patients	• Approximately 300 repeat screening customers
• Hospital care line		• Blood pressure medications adjusted for approximately 36 customers
		• Several dozen referrals to hospital care line
		**Future goals**
		• Collaborate with engineers to develop a secure database for electronically charting customer data across a whole season
		• Contribute to preventive lifestyles for community members
		• Track relationship between regular screening/health-related consultation and health service use/costs
**Standard 6: Measure and improve performance**		
• Annual survey of market customers	• Survey and community focus group assesses customer use of market, perception of existing market services, and suggestions for future improvement	**Current outcomes **
		• Surveys indicated customer demographics, modes of transportation to market, prevalence of outstanding medical conditions in customers (ie, high blood pressure, high cholesterol, arthritis), and greater demand for organic produce
• Community focus group to improve market services		
	• Faculty members provided student mentorship in grant writing to expand market services	
• Approximately 10 faculty mentors		
		• Provided grant-writing and leadership opportunities for students
		• 3 grants submitted and 2 funded
		**Future goals **
		• Participate in national Prescription Produce program
		• Comprehensive database of market inputs, outputs, and outcomes related to patient-centered medical home standards

^a^ Data from Facebook (22).

## Interpretation

We have found that medical center markets can uniquely and cost-effectively support a medical center in achieving NCQA standards of the PCMH. The whole-person focus of the PCMH model demands not only competent clinical care but also commitment to such concepts as sensitivity to context and cultural differences, interdisciplinary collaboration, community engagement, and access to care — all of which can be addressed in the community space of a market. Markets that are developed around local needs and operate recurrently for extended periods may be especially valuable in areas that have a high prevalence of chronic disease. Collaborative partnerships between medical centers and markets could promote PCMH goals in multiple geographic regions and help train current and future health professionals to provide comprehensive, patient-centered care.
